# Evaluation of the effectiveness of sleep hygiene education and FITBIT devices on quality of sleep and psychological worry: a pilot quasi-experimental study among first-year college students

**DOI:** 10.3389/fpubh.2023.1182758

**Published:** 2023-08-23

**Authors:** Wegdan Bani Issa, Heba Hijazi, Hadia Radwan, Roba Saqan, Alham Al-Sharman, A. B. Rani Samsudin, Randa Fakhry, Nabeel Al-Yateem, Rachel C. Rossiter, Ali Ibrahim, Ibrahim Moustafa, Farah Naja, Mohamad Alameddine, Nada Abbas, Dana N. Abdelrahim, Arwa Al-Shujairi, Manal Awad

**Affiliations:** ^1^College of Health Sciences, University of Sharjah, Sharjah, United Arab Emirates; ^2^Faculty of Medicine, Jordan University of Science and Technology, Irbid, Jordan; ^3^Research Institute of Medical and Health Sciences University of Sharjah, Sharjah, United Arab Emirates; ^4^College of Dental Medicine, University of Sharjah, Sharjah, United Arab Emirates; ^5^School of Nursing, Paramedicine and Healthcare Sciences, Faculty of Science and Health, Charles Sturt University, Bathurst, NSW, Australia; ^6^College of Business and Economics, United Arab Emirates University, Abu Dhabi, United Arab Emirates; ^7^Department of Nutrition and Food Sciences, American University of Beirut, Beirut, Lebanon; ^8^Research Institute of Medical and Health Sciences, University of Sharjah, Sharjah, United Arab Emirates; ^9^Glaxosmithkline, Dubai, United Arab Emirates; ^10^University Dental Hospital Sharjah, College of Dental Medicine, University of Sharjah, Sharjah, United Arab Emirates

**Keywords:** college students, sleep quality, sleep hygiene, FITBIT, actigraph sleep trackers

## Abstract

**Background:**

College students report disturbed sleep patterns that can negatively impact their wellbeing and academic performance.

**Objectives:**

This study examined the effect of a 4-week sleep hygiene program that included sleep education and actigraph sleep trackers (FITBITs) on improving sleep quality and reducing psychological worry without control group.

**Design, settings, and participants:**

A pilot quasi-experimental design, participants were randomly selected medical and health sciences from a university students in the United-Arab-Emirates.

**Methods:**

Students were asked to wear FITBITs and log their daily sleep data and completed the Pittsburgh Sleep Quality Index (PSQI) and Penn State Worry Questionnaire (PSWQ). Extensive sleep hygiene education was delivered via lectures, a WhatsApp group, and the Blackboard platform. In total, 50 students completed pre-and post-assessments and returned FITBIT data.

**Results:**

There was a significant difference in the prevalence of good sleep postintervention compared with pre-intervention (46% vs. 28%; *p* = 0.0126). The mean PSQI score was significantly lower post-intervention compared with pre-intervention (6.17 ± 3.16 vs. 7.12.87; *p* = 0.04, Cohen’s d 0.33). After the intervention, subjective sleep quality, sleep latency, and daytime dysfunction were significantly improved compared with pre-intervention (*p* < 0.05). In addition, FITBIT data showed total sleep time and the number of restless episodes per night were significantly improved postintervention compared with pre-intervention (*p* = 0.013). The mean PSWQ score significantly decreased from pre-intervention to *p* = 0.049, Cohen’ d = 0.25. The correlation between PSQI and PSWQ scores was significant post-intervention (β = 0.40, *p* = 0.02).

**Conclusion:**

Our results may inform university educational policy and curricular reform to incorporate sleep hygiene awareness programs to empower students and improve their sleep habits.

## 1. Introduction

Good sleep quality is important for physical, mental, and cognitive functioning and could impact the quality of life of all age groups (1). Chronic sleep disturbance has become a public health concern that is linked to a number of serious medical conditions, including cancer, obesity, stroke, dementia, hypertension, metabolic disorders, and cardiovascular diseases. Adults should sleep 7–9 h per night to prevent the long-term health consequences of poor sleep on quality of life and daily (2). Throughout different life stages, people are subjected to alternations in their sleeping patterns and habits. College students are at increased risk for poor sleep quality, which is attributed to the transition to new experiences and academic life (3, 4). Especially vulnerable to disturbed sleeping hygiene are students in demanding specialties, such as Medicine and Health Sciences (3, 4). Although the transition to college can be exciting for students and their families, students face new challenges and expectations associated with college life and require effective coping abilities (3, 4). Different reports from various countries emphasized that poor sleep is a common problem among college students, with an overall prevalence rate of more than 50% (5).

It is also common for college students to report symptoms of psychological worry related to stressors associated with academic life, such as achieving good grades, submitting projects/assignments, and mastering large amounts of content in a limited time, in addition to other life stressors and future career expectations (6). Psychological worry symptoms (e.g., over thinking, feeling nervous, inability to concentrate, feeling apprehensive, and being hyper alert) that result in excessive worry and development of generalized anxiety disorder (GAD) are common among students studying medicine and health-related disciplines (6, 7). Evidence suggests that poor sleep is connected to psychological worry in college students (7). Lack of sleep makes it harder for students to concentrate, and function throughout the day; therefore, they may develop pathological worry, which is a primary manifestation of GAD (8). Students with poor sleep quality were reported to be 4.7-times more likely to have higher psychological worry symptoms than students with good sleep quality (9). This association became more significant when students were faced with deadlines related to academic tasks (e.g., exams/projects and assignments) (6). It has also been suggested that the association between sleep and worry is bidirectional, as psychological worry may lead to poor sleep quality and insomnia (10, 11).

Although sleep deprivation is associated with negative outcomes among college students, including psychological worry symptoms, this problem has not been adequately addressed in this population. Most available studies among first-year college students involved the subjective assessment of sleep quality, and few studies included programs to improve sleep quality (12). Poor sleep quality and psychological worry are substantial problems for first-year students, especially those enrolled in medicine and health-related disciplines because of new transitional experiences and high competition with persistent pressure to achieve a better academic standing. In the United Arab Emirates (UAE), no studies have tackled sleep problems among first-year students from medical colleges or explored integrated interventions that may improve their sleep and worry symptoms. There is an urgent need for interventions that improve sleep quality and reduce the impact of poor sleep on students’ daily functioning and wellbeing (13). The primary aim of this pilot quasi-experimental study was to evaluate the effectiveness of a tailored sleep educational program that included sleep education and sleep tracking (FITBIT) devices and evaluate sleep quality using both subjective (Pittsburgh Sleep Quality Index; PSQI) and objective (FITBIT) sleep data. The secondary aim was to examine the effect of the sleep educational program on the level of psychological worry among first-year medical and health sciences students from one university in the UAE. Furthermore, we examined the association between sleep quality and psychological worry following the intervention to offer recommendations for further intervention studies.

## 2. Materials and methods

### 2.1. Study design

This study used a pilot quasi-experimental design with no control group. Subjective and objective measures were used to collect information on sleep quality and psychological worry from participants over a 4-week period. The recruitment process extended over 3 months (September to November 2020).

### 2.2. Study population and sampling

The target population was first-year students admitted to the medical and health sciences colleges at the study university for the 2020/2021 academic year. First-year students were chosen because this is considered a transitional period to academic life with new expectations, and coping mechanisms. A list of classes with students enrolled in medicine and health sciences in the fall of the 2020/2021 academic year was accessed from the university’s registration department. We randomly selected 10 classes from the list with an average class size of 30 students. All students enrolled in these classes were invited to participate through an invitation letter shared via Blackboard (web-based course-management system used by the university) and directly during regular classes on campus.

The expected sample size was calculated using G-power software assuming 90% power (β = 10%), alpha of 5% (type 1 error), and an effect size of 0.5 for paired t-tests. This showed that a sample of 44 students was required (14). We invited 200 students from the selected classes to participate to compensate for expected missing data (especially FITBIT sleep data) and attrition. We included female and male students from any nationality, who did not suffer from any chronic or mental health problems and attended regular first-year classes. Students who reported having psychiatric problems or sleeping disorders or that were taking prescribed sleeping medications were excluded from this study.

### 2.3. Recruitment method

**Figure 1:**
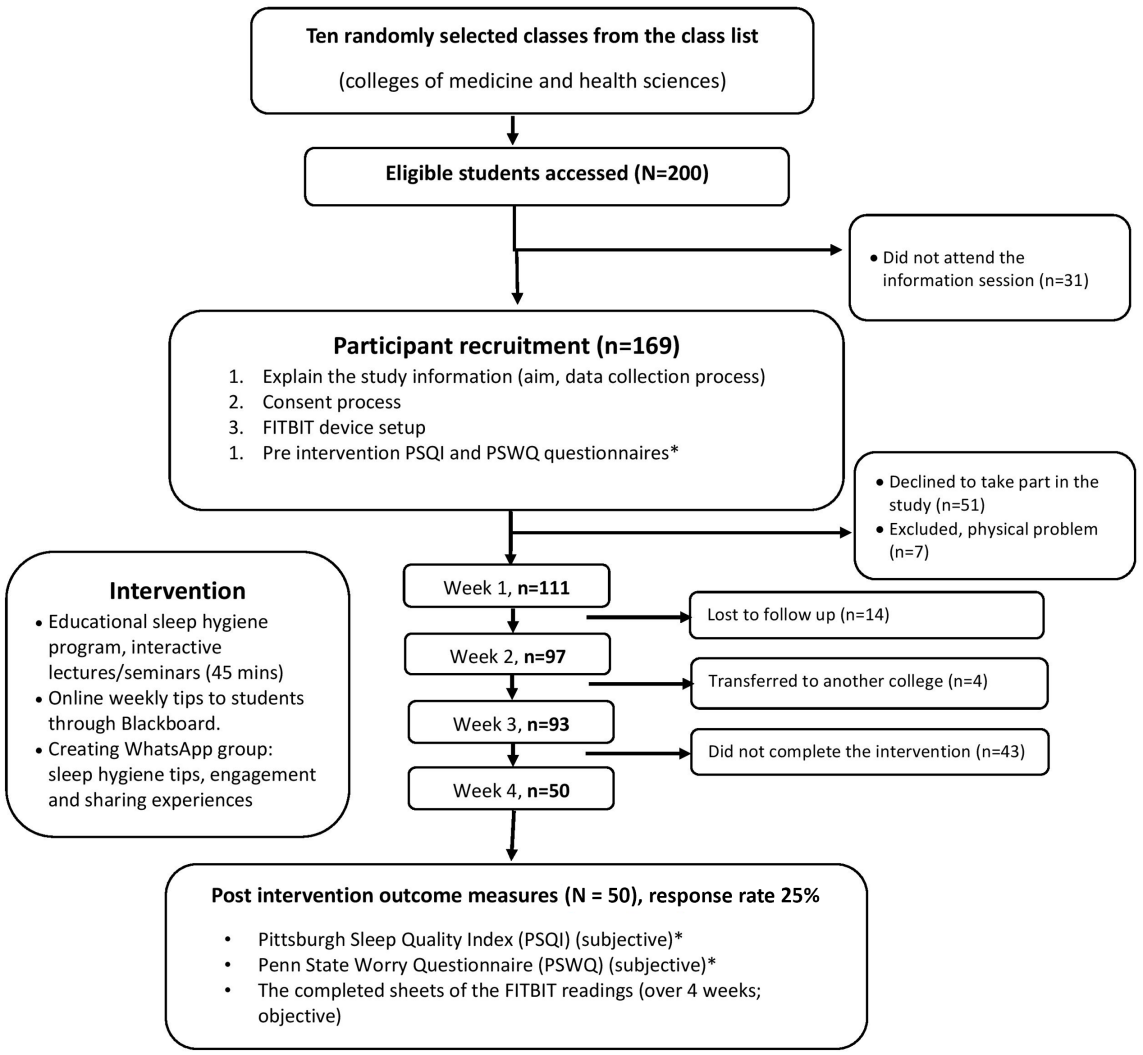
Flowchart for data collection process.

Following random selection of classes, instructors of these classes were approached to invite their students to participate in this study through announcements on the Blackboard platform and during regular classes. The research team visited the selected classrooms for which course instructors had granted access and explained the study, data collection process, and provision of consent (Figure 1).

Eligible students who agreed to participate and provided informed consent were asked to complete the study questionnaire, which included demographic information, the PSQI and the Penn State Worry Questionnaire (PSWQ). Next, students were instructed to wear a FITBIT all of the time for a 4-week period and record their daily sleep data from the FITBIT on a written sheet. Students were instructed about installing and registering the FITBIT software on their smartphones. Data recorded from the FITBITs covered sleeping parameters per night, number of hours slept, number of deep sleep hours, number of minutes to fall asleep, number of times awake, number of times restless, and effective hours of sleep. After completion of the 4-week period, participants were asked to complete the PSQI and PSWQ a second time and return the daily sleep tracking sheets extracted from the FITBITs.

### 2.4. The intervention: sleep hygiene education program

The intervention comprised a sleep education program that was based on best sleep hygiene behaviors designed to improve sleep quality. Sleep hygiene refers to “…a variety of different practices that are necessary to have normal, quality nighttime sleep and full daytime alertness” (15). The program was prepared after an extensive review of relevant literature (15–17) and consisted of weekly interactive seminars/lectures on sleep hygiene education (total four lectures). Furthermore, sleep hygiene tips were distributed during classes (via brochures) and through Blackboard and WhatsApp groups. The program was delivered by two sleep specialists and a trained research team to ensure the quality and consistency of information delivered to participants.

The sleep education program content included information about the physiology of sleep, effects of sleep on health and wellbeing on college students, best sleep hygiene practices/tips, and consequences of poor sleep (see Table 1). Students who reported having chronic sleep deprivation were referred to sleep specialists who worked at a hospital affiliated with the university. To motivate participants to continue in the study, the research team created a WhatsApp group to send daily messages about good sleep habits. Students were also encouraged to share experiences about sleep habits on WhatsApp to learn about best sleep practices from each other. In addition, course instructors were requested to send daily sleep hygiene tips via Blackboard for students to remind them to adhere to good sleep practices and keep wearing the FITBIT and recording sleep data on the sheet provided.

**Table 1:** List of sleep hygiene educational program components and recommendations.

Sleep hygiene behavioral components	Recommendations
Bedrooms and sleep surroundings	Have a quiet, comfortable bedroom
	Set your bedroom thermostat at a comfortable temperature (a little cooler is better)
	Make sure you have clean fresh air in the room
	Bedroom should be dark and quiet, and slightly cool
Sleep routine	Have a comfortable pre-bedtime routine (e.g., a warm bath or shower, meditation, or quiet time)
	Maintain a regular sleep time routine
	Go to bed at the same time
	Wake up at the same time
	Do not stay in bed awake for more than 5–10 min (i.e., get out of bed if you cannot sleep)
	Do not read, write, eat, watch TV, talk on the phone, or play cards in bed
	Limit daytime naps
Caffeine, nicotine, and food consumption	Cut down on caffeine
	If you drink caffeine, consume it only before noon
	Quit smoking, if you could not try to avoid cigarettes at bedtime
	Do not go to bed hungry, but do not eat a big meal near bedtime either
Screen-time exposure	Do not watch TV or use a computer or smartphone while in bed
	Avoid exposure to screens 2 h before bedtime
Exercising	Exercise regularly (i.e., every day)
	Exercising is recommended before 2:00 p.m.
	Avoid rigorous exercise before bedtime

### 2.5. Outcome measures

#### 2.5.1. Demographics and general information sheet

The general information part of the questionnaire included questions about participants’ basic demographic information, including age, gender, nationality, residency, college, major, medical conditions, living arrangements (inside or outside the students’ dormitory), parents’ education level, smoking status, screen exposure time (hours), and frequency of use of social media platforms (Facebook, YouTube, WhatsApp, Instagram, TikTok, Snapchat, Pinterest, LinkedIn, and Twitter).

#### 2.5.2. Self-report questionnaires

##### 2.5.2.1. PSQI

The PSQI is a well-known self-report questionnaire that assesses the overall quality of sleep over the last month (18). It comprises 19 items that differentiate “poor” from “good” sleepers by measuring seven sleep domains: Subjective sleep quality, sleep latency, sleep duration, habitual sleep efficiency, sleep disturbances, use of sleep medication, and daytime dysfunction. The global PSQI score ranges from 0 to 21, and a higher score is indicative of worse sleep quality (score > 5 indicates poor sleep quality) (18, 19). The PSQI has been widely used with sensitivity of 89.6% and specificity of 86.5% in differentiating good from poor sleepers (19). Furthermore, a good level of internal consistency between the items was reported (Cronbach’s alpha 0.83). The PSQI has been used in a similar population to this study and reported to be acceptable with sufficient reliability indices (19–21).

##### 2.5.2.2. PSWQ

The PSWQ is a self-reported questionnaire designed to measure the trait of psychological worry, which is the main feature of GAD. The PSWQ comprises 16 items that measure the excessiveness/intensity, generality, and uncontrollable dimensions of psychological worry (22). The PSWQ has been validated in college student (22, 23) and clinical samples (23). Responses are on a Likert rating scale from 1 (not at all typical of me) to 5 (very typical of me). Scores range from 16 to 80, with high scores (60–80) indicating high levels of worry, scores of 40–59 indicating moderate worry, and scores of 16–39 indicating a low level of worry (22). PSWQ scores were positively correlated with other measures of pervasive worry, which supported the discriminate validity of the tool (24). Moreover, the PSWQ was reported to have high internal consistency and good test–retest reliability (22).

#### 2.5.3. Sleep activity tracker (FITBIT)

The FITBIT flex device was used to track participants’ sleep activity (25, 26). The accelerometer synchronizes with the manufacturer’s software on mobile or computer devices to track physical activity and total hours slept, sleep latency, and an “arousal index” based on episodes of movement during presumed sleep time. The FITBIT device is the most widely used and was found to have high inter-device reliability (96.5–99.1), the ability to detect sleep duration and efficiency, and was acceptable by subjects (27).

Sleep parameters were detected using the FITBIT device over a 4-week period and recorded (collected) on a written sheet that was given to participants. Based on the FITBIT data, students were requested to record sleep information each day, including the number of hours slept, number of deep sleep hours, number of minutes to fall asleep, number of times awake, number of times of restlessness, and effective hours of sleep. Restless sleep indicated that the participants had moved a lot during sleep, such as tossing and turning. Actual time asleep was when the body was at rest completely and had not moved (see more information on FITBIT at houritbit.com/flex). Moreover, considering the variation in sleep patterns throughout weekdays and weekends, participants were asked to record their sleep diary using the provided sheet during weekdays and weekends.

### 2.6. Ethical approval

All procedures performed in this study were in accordance with the ethical standards followed by the World Medical Association’s Declaration of Helsinki and the principle investigator’s institution and approved by the Research Ethics Committee (REC/15/12/10). Participation was completely voluntary, and participants were given the choice to refuse participation or dropout of the study without consequences, especially related to their academic life and grades.

### 2.7. Statistical analyses

Descriptive statistics were presented as means and standard deviations for continuous variables and frequencies and percentages for categorical variables. Differences between pre- and post-intervention were tested using paired sample *t*-tests or non-parametric Wilcoxon signed-rank tests, depending on data normality. Repeated measures analysis of variance was used to test for any differences in mean indicators at four time points (weeks 1, 2, 3, and 4); if significant, a *post hoc* pairwise comparison was run. Bivariate correlation was used to determine correlations between PSQI and PSWQ scores (standardized beta coefficient). Cohen’s d was used to estimate the effect size. SPSS version 26 (SPSS Inc., Chicago, IL) was used for all statistical analyses. *p*-values < 0.05 were considered statistically significant.

## 3. Results

### 3.1. General characteristics of the study population

Table 2 summarizes the characteristics of the study population. In total, 200 first-year students were invited to participate, and 50 students completed the study and returned all surveys and sleep tracking sheets (FITBIT data; response rate of 25%). Participants were dismissed from the study for a variety of reasons, including refusal to participate in the study and failure to respond to or follow up during various study phases (see Figure 1). Participants’ mean age was 17.93 ± 0.296 years. Most participants were female (82%) and 60% were non-Emirati Arabs. The majority of students lived off campus (72%) and about half were residing in Sharjah (54%). Participants were first-year students from two colleges: The College of Health Sciences (60%) and the College of Medicine (40%). Most participants’ parents had a university-level education (70 and 48% for fathers’ and mothers’ education; respectively). Twenty-five participants (50%) reported spending more than 5 h of screentime daily; 30% reported using Instagram and 50% used two or more social media types.

**Table 2:** Basic sociodemographic characteristics of the study population (*N* = 50).

Variable		*n*	%
Age (mean 17.93 ± 0.296 years)			
Gender	Male	9	18.0
	Female	41	82.0
Nationality	Arab	30	60.0
	Non-Arabs	8	16.0
	Emirati	12	24.0
Area of residency	Sharjah	27	54.0
	Ajman	8	16.0
	Dubai	9	18.0
	Abu-Dhabi	2	4.0
	Al-Ain	0	0.0
	Ras-Al-Khaimah	2	4.0
	Al-Fujairah	2	4.0
School	College of Health Sciences	30	60.0
	College of Medicine	20	40.0
Accommodation	Living on campus	14	28.0
	Living off campus	36	72.0
Fathers’ education	Elementary	5	10.0
	High school	10	20.0
	University	35	70.0
Mothers’ education	Elementary	9	18.0
	High school	17	34.0
	University	24	48.0
Number of hours spent on computer, phone, or laptop	<3	6	12.0
	3–4	19	38.0
	5+	25	50.0
Social media platforms most frequently used	Facebook	2	4.0
	Instagram	15	30.0
	Snapchat	3	6.0
	Twitter	3	6.0
	Two or more	25	50.0
	None of the above	2	4.0
Smoking	Yes	5	10.0
	No	45	90.0
Overall sleep quality (baseline)	Good sleep quality (PSQI < 5)	14	28.0
	Poor sleep quality (PSQI ≥ 5)	36	72.0

### 3.2. Participants’ sleep quality using subjective report (PSQI) and objective data (FITBIT)

**Figure 2:**
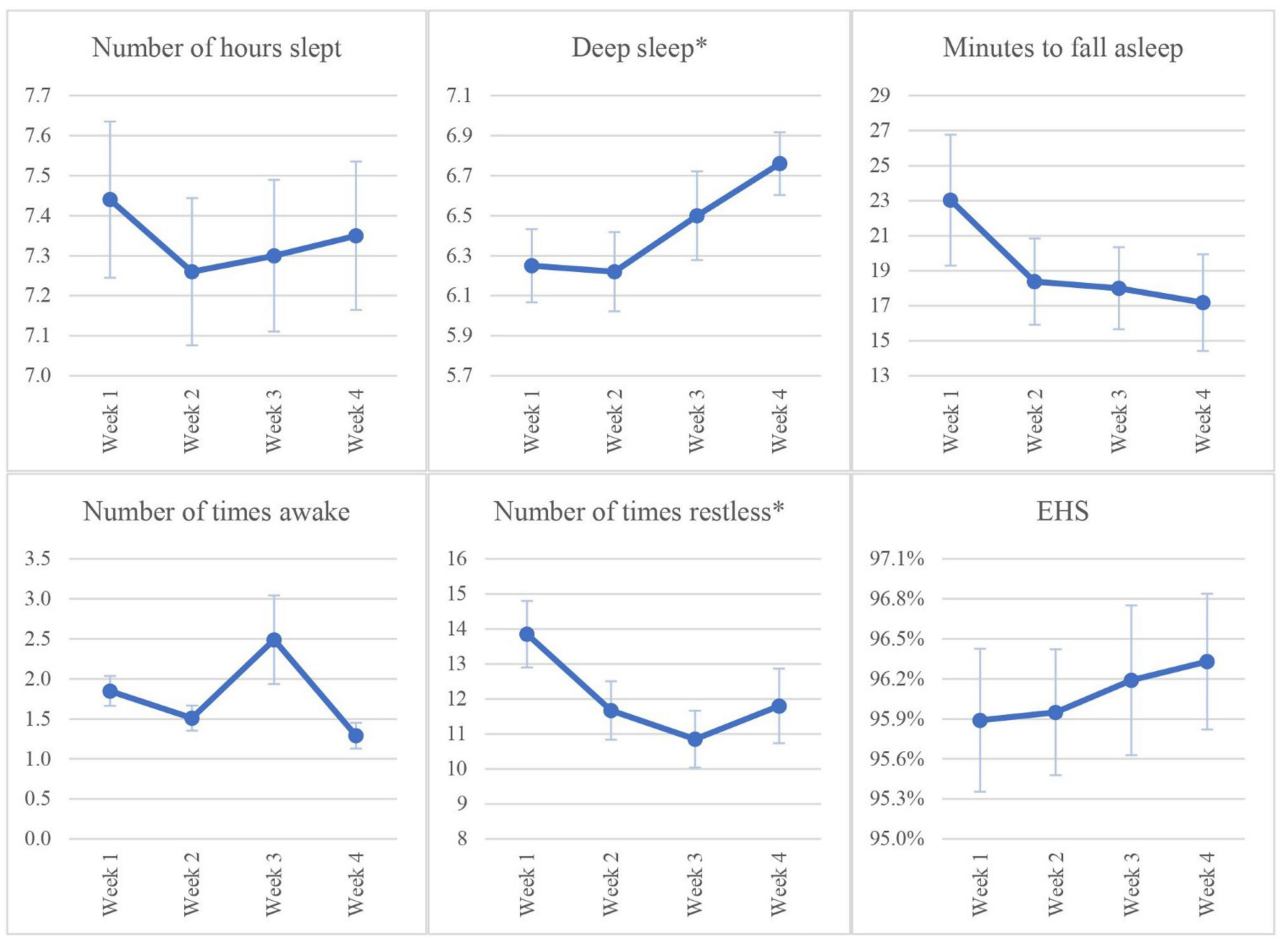
Changes in FITBIT tracker data at weeks 1, 2, 3, and 4. Number of hours slept = time awake – time asleep. EHS (Effective Hours Slept) = Number of hours slept/(Number of hours slept + Minutes to fall asleep)* 100. Values are plotted as mean ± standard error. *p < 0.05.

The prevalence of poor sleep before and after the intervention is presented in Table 3. There was a significant increase in the number of students who reported good sleep post-intervention (46%) compared with baseline (28%; *p* = 0.0126). Table 4 presents participants’ PSQI scores before and after the intervention. The mean PSQI total score was significantly lower post-intervention than pre-intervention (6.17 ± 3.16 vs. 7.1 ± 2.87; *p* = 0.04, Cohen’s d 0.33) indicating better overall sleep quality after the intervention. The PSQI scores for three sleep components were significantly lower post-intervention compared with pre-intervention, which also confirmed better sleep quality post intervention: Subjective sleep quality (component 1; *p* = 0.008), sleep latency (component 2; *p* = 0.006), and daytime dysfunction (component 7; *p* = 0.040). Changes in FITBIT tracker data over weeks 1, 2, 3, and 4 are presented in Table 5 and Figure 2. Two sleep parameters were significantly improved post-intervention compared with pre-intervention: The number of deep sleep hours per night (*p* = 0.002) and number of times of restlessness per night (*p* = 0.013).

**Table 3:** Prevalence of good sleep quality among students pre- and post-intervention using the Pittsburgh Sleep Quality Index (*N* = 50).

	Good sleep quality	Poor sleep quality	*p*-value
Pre-intervention	14 (28%)	36 (72%)	0.0126^*^
Post-intervention	23 (46%)	27 (54%)	

*Based on chi-square test. *p*-value was significant at 0.05.

**Table 4:** Pittsburgh Sleep Quality Index (PSQI) components and total score at baseline and post-intervention (*N* = 50).

	Pre-intervention (baseline)	Post-intervention (endline)	*p*-value
	Mean ± SD	Mean ± SD	
PSQI Component 1: subjective sleep quality	0.34 ± 0.69	0.08 ± 0.34	**0.008**
PSQI Component 2: sleep latency	1.33 ± 1.13	1.02 ± 0.88	**0.006**
PSQI Component 3: sleep duration	1.49 ± 1.06	1.47 ± 0.98	0.896
PSQI Component 4: habitual sleep efficiency	0.76 ± 1.01	0.49 ± 0.94	0.093
PSQI Component 5: sleep disturbances	1.35 ± 0.56	1.20 ± 0.58	0.181
PSQI Component 6: use of sleeping medication	0.47 ± 0.65	0.65 ± 0.93	0.228
PSQI Component 7: daytime dysfunction	1.63 ± 0.67	1.18 ± 0.78	**0.001**
^*^PSQI total score	7.1 ± 2.87	6.17 ± 3.16	**0.040**

*Significant at *p* value 0.05. Each component has a range of 0 to 3 points. A score of “0” indicates no difficulty, whereas a score of “3” indicates severe difficulty. The seven component scores are then added to yield the total score, with a range of 0 to 21 points, with “0” indicating no difficulty and “21” indicating severe difficulties. SD, standard deviation.

**Table 5:** Changes in FITBIT tracker data at weeks 1, 2, 3, and 4 (*N* = 50).

	Number of hours slept per night^*^	Number of deep sleep hours per night	Number of minutes to fall asleep per night	Number of times awake per night	Number of restless times per night^**^	EHS per night^***^
Week 1	7.44 ± 1.38	6.25 ± 1.29	23.03 ± 26.44	1.85 ± 1.32	13.85 ± 6.72	95.89% ± 3.79
Week 2	7.26 ± 1.3	6.22 ± 1.4	18.38 ± 17.43	1.51 ± 1.11	11.67 ± 5.9	95.95% ± 3.34
Week 3	7.3 ± 1.34	6.5 ± 1.57	18 ± 16.57	2.49 ± 3.92	10.85 ± 5.74	96.19% ± 3.97
Week 4	7.35 ± 1.31	6.76 ± 1.11	17.18 ± 19.51	1.29 ± 1.14	11.8 ± 7.53	96.33 ± 3.6
*F*-statistics	1.663	3.807	1.935	1.369	4.724	0.546
*p*-value	0.209	0.022	0.177	0.263	0.013	0.594

^*^Number of hours slept = time awake − time asleep. ^**^Number of restless times means number of times awake. ^***^EHS (effective hours slept) = number of hours slept/(number of hours slept + minutes to fall asleep) × 100.

### 3.3. Participants’ levels of psychological worry (PSWQ)

Results of the PSWQ are summarized in Table 6. Participants’ psychological worry level was significantly decreased post-intervention compared with pre-intervention (50.51 ± 12.23 vs. 47.12 ± 13.33; *p* = 0.049, Cohen’s d = 0.25). Statistically significant changes were noted in items 2, 6, and 16 (“My worries overwhelm me,” “When I am under pressure. I worry a lot,” and “I worry about projects until they are all done”).

**Table 6:** Penn State Worry Questionnaire (PSWQ) components and total score at baseline and post-intervention (*N* = 50).

	Pre-intervention (baseline)	Post-intervention (endline)	*p*-value
	Mean ± SD	Mean ± SD	
PSWQ 1: If I do not have enough time to do everything, I do not worry about it	3.49 ± 1.26	3.41 ± 1.19	0.719
PSWQ 2: My worries overwhelm me	3.39 ± 1.19	2.80 ± 1.24	0.006
PSWQ 3: I do not tend to worry about things	3.57 ± 1.32	3.67 ± 1.2	0.620
PSWQ 4: Many situations make me worry	3.08 ± 1.22	2.71 ± 1.22	0.074
PSWQ 5: I know I should not worry about things, but I just cannot help it.	3.14 ± 1.38	2.90 ± 1.31	0.274
PSWQ 6: When I am under pressure, I worry a lot	3.59 ± 1.24	2.96 ± 1.32	0.004
PSWQ 7: I am always worrying about something.	2.98 ± 1.25	2.67 ± 1.25	0.100
PSWQ 8: I find it easy to dismiss worrisome thoughts.	3.27 ± 1.06	3.45 ± 1.21	0.322
PSWQ 9: As soon as I finish one task, I start to worry about everything else I have to do.	2.67 ± 1.25	2.61 ± 1.29	0.781
PSWQ 10: I never worry about anything.	4.04 ± 1.17	3.69 ± 1.21	0.107
PSWQ 11: When there is nothing more I can do about a concern, I do not worry about it anymore.	3.18 ± 1.09	3.00 ± 1.19	0.310
PSWQ 12: I have been a worrier all my life.	2.37 ± 1.09	2.67 ± 1.21	0.145
PSWQ 13: I notice that I have been worrying about things.	3.14 ± 1.21	2.84 ± 1.18	0.149
PSWQ 14: Once I start worrying, I cannot stop.	2.84 ± 1.37	2.80 ± 1.26	0.842
PSWQ 15: I worry all the time.	2.33 ± 1.16	2.61 ± 1.13	0.099
PSWQ 16: I worry about projects until they are all done.	3.43 ± 1.31	2.94 ± 1.3	0.020
PSWQ total score	50.51 ± 12.23	47.12 ± 13.33	0.049

In scoring the PSWQ, a value of 1, 2, 3, 4, and 5 was assigned to a response depending upon whether the item was worded positively or negatively. The total score of the scale ranges from 16 to 80. Items 1, 3, 8, 10, 11 are reverse scored. A lower score is indicative of a positive outcome. SD, standard deviation.

### 3.4. Association between sleep and worry scores

After the intervention, a significant correlation was observed between total PSQI scores and PSWQ scores (β = 0.40, *p* = 0.02), whereas the correlation pre-intervention was non-significant (β = 0.198, *p* = 0.19).

## 4. Discussion

This study emphasized the importance of sleep hygiene education to promote healthy sleep patterns and decrease psychological worry among college students. The findings of this study can be used to re-evaluate and reconsider the role of educational institutions in integrating early sleep interventions among first-year students to minimize the negative consequences of poor sleep on their health and academic performance. Consistent with previous studies, we found 72% of participants reported poor sleep, confirming that poor sleep is a global problem among college students (5, 28, 29). Our results were consistent with previous studies from different countries in which students reported poor sleep quality, including Germany (30, 31), the US (31, 32), Italy (5) India (12), and China (33).

Interestingly, the prevalence of poor sleep quality based on subjective report (PSQI scores) in our study decreased after the sleep education intervention. These results may be indicative of the positive impact of sleep hygiene education on improving sleep quality and habits among college students. Our results were consistent with previous studies that used similar interventions to promote sleep among college students (34, 35). Post-intervention, participants reported improvement in three PSQI sleep components: Sleep latency, daytime dysfunction, and subjective sleep.

These results indicated that students spent less time in bed before falling asleep and had more effective night sleep (34). It is possible that the sleep intervention component related to decrease screen time while in bed was efficient in decreasing the time participants needed to fall asleep. Increased screen time has been identified as an important factor that leads to increased sleep latency in an adolescent population (20). In addition, improving daytime dysfunction means that participants felt more active and less tired during the day as a result of having a good sleep (36).

Changes in both sleep latency and daytime functioning may indicate that the sleep education program increased students’ awareness about importance of sleep for their health. A cross-sectional study involving undergraduate students from the US reported a significant correlation between sleep valuation (subjective perception of sleep) and sleep outcomes (37). Furthermore, improvement in the subjective sleep quality component of the PSQI confirmed that students appreciated the value of sleep after the intervention in our study. These results were also consistent with prior research that demonstrated improvement in subjective reports of sleep among college students following sleep intervention (34, 35). Self-perception of the value of sleep is critical to initiate change in sleep behavior, which is also connected to better daytime functioning and less daytime napping (38). This suggested that increasing sleep knowledge may be beneficial for college students, especially medical students who have heavy study loads (39). The persistent approach we used involving a WhatsApp discussion group and the Blackboard platform appeared to be an efficient way to induce changes in sleep habits in our population. The sharing of sleep experiences through WhatsApp conversation and frequent reminders might also have assisted in maximizing the benefits of the sleep education program. Increased knowledge about sleep is essential to impact change in sleep behaviors among students (39). Future studies may include measures to evaluate the effectiveness of using different interactive media and digital programs in promoting sleep among college students.

Improvement in sleep after the intervention in our study was confirmed objectively by the FITBIT data. FITBIT data showed a significant increase in deep sleep hours and decrease in number of restless times, which reflected improvement in participants’ sleep patterns. These results were consistent with prior research where use of a FITBIT assisted in creating daily sleep diary and encouraged students to improve their sleep profile (30). Among 692 undergraduate students at the University of Notre Dame, participants who trusted FITBIT data reported adjustment in their sleep habits (40). A US study that used the FITBIT as a sleep tracker to measure sleep among 100 students in an introductory college chemistry class (88 of whom completed the study) found it correlated well with academic performance (41). Use of FITBITs offers a way to longitudinally and automatically quantify sleep quality with little effort needed (30, 42). However, in our population, the compliance rate was less than expected, which could be attributable to lack of trust in FITBIT data. Students must be encouraged to continue wear the FITBIT and consider it as valuable sleep tracking device so they change their sleep habits.

The sleep intervention program used in the present study coincided with a decrease in psychological worry, as evidenced by the lower total PSWQ scores post-intervention. It was found that individuals with shorter sleep duration and who had sleep disturbance were more likely to develop worry symptoms such as intrusive thoughts, cognitive arousal, and persistent and negative thoughts leading to mental illness and anxiety symptoms (43–45). Our results related to the improved association between psychological worry and sleep quality scores after the intervention further emphasized the connection between sleep and worry symptoms (43–46). It is possible that good sleepers in our population had better confidence and control of their daily activities and performance, better mental focus, and higher academic achievement, and therefore had less worry symptoms than those with poor sleep. However, the correlation between sleep and psychological worry needs further exploration (46). Understanding this relationship has potential to elucidate key factors involved in these phenomena and may aid in the development and implementation proper interventions. It is suggested to use dairies to determine how sleep leads to worry and at what time of sleep (7).

In our study, we used multiple approaches that appeared to be suitable for college students, including classroom teaching and online strategies (interactive WhatsApp groups and the Blackboard platform) combined with interactive technology (education and FITBITs). The use of lectures, online teaching (47), or psychological therapies (cognitive behavioral therapy) (48, 49) alone are not sufficient to create change in sleep behaviors among students. However, the use of interactive technology along with theoretical education about sleep appeared to be efficient in changing sleep habits (50). It is recommended that personalized interventions are used as each student’s sleep schedule and habits may be different (48, 49).

It is important to acknowledge that we attempted to recruit students to serve as a control group, but unfortunately most returned data were incomplete and therefore data analysis could not be processed. Because of a limited budget to buy more FITBIT devices, we could not replace these missed participants.

The limitation of the quasi – experimental design related to lacking a control group, lack of control of extraneous variables must be acknowledged and may limit the ability to generalize from the results of the current study. It is important that further studies measure the sustainable impact of an intervention for sleep habits among students at an introductory level. Special attention needs to be given to possible mediators such as previous knowledge of sleep habits, the extent of utilizations of digital screens in bed, and the parental role in promoting sleep hygiene practices. In future study we will continue refine the methodologies to understand the complex relationship between sleep interventions programs, mediators and outcome.

## 5. Conclusion

In conclusion, we found that sleep hygiene education and FITBIT devices can significantly enhance sleep quality as measured by subjective and objective data. In addition, this education may help improve students’ psychological worry, which will help minimize the development of mental illness and enhance their daily performance. Longitudinal studies are recommended to determine the lasting effect of sleep interventions in changing sleep behavior among college students. Input from students may be helpful in developing proper sleep interventions for college students. Sleep intervention is essential to enhance students’ awareness about the importance of adopting healthy sleep habits to sustain their wellbeing. Educational policies should consider sleep as a priority for first-year college students. Students must be supported to adopt healthy sleep habits that improve their mental and physical health and academic performance.

## Data availability statement

The original contributions presented in the study are included in the article/supplementary material, further inquiries can be directed to the corresponding author.

## Ethics statement

The studies involving human participants were reviewed and approved by University of Sharjah Research Ethics Committee approval number (REC/15/12/10). The patients/participants provided their written informed consent to participate in this study.

## Author contributions

WB, HH, HR, RS, AlA-S, AS, RF, NA-Y, RR, AI, IM, FN, MAl, NA, DA, ArA-S, and MAw conceived this study, contributed to data collection and analysis, interpreted data, and drafted the manuscript. WB, HH, HR, RS, AlA-S, AS, RF, NA-Y, RR, AI, IM, FN, MAl, and MAw contributed to data collection. WB, RS, DA, and NA contributed to data analysis. WB, NA, DA, ArA-S, and MA conceived this study. WB, HH, HR, RS, AlA-S, DA, and MA contributed to drafting the manuscript. WB, HH, HR, RS, AlA-S, RF, NA-Y, RR, AI, IM, FN, DA, and MAw contributed to writing the manuscript. All authors contributed to the article and approved the submitted version.

## Funding

This project is funded by Health Promotion Research Group/ Research Institute of Medical and Health Sciences, University of Sharjah, United Arab Emirates, Grant/Award Number: 150310.

## Conflict of interest

ArA-S was employed by Glaxosmithkline.

The remaining authors declare that the research was conducted in the absence of any commercial or financial relationships that could be construed as a potential conflict of interest.

## Publisher’s note

All claims expressed in this article are solely those of the authors and do not necessarily represent those of their affiliated organizations, or those of the publisher, the editors and the reviewers. Any product that may be evaluated in this article, or claim that may be made by its manufacturer, is not guaranteed or endorsed by the publisher.
